# Parental Corrections

**Published:** 1863-11

**Authors:** 


### PARENTAL CORRECTIONS:

WITH the following illustration, we conclude the subject treated of in the two preceding numbers, with the earnest desire that they may exert a wholesome and restraining influence on the minds of parents, in reference to hasty and tyrannical punishments of their little ones, and thus save them from unavailing sorrows, and vain regrets and eating remorses, at a later period of life, when unavoidable troubles come fast enough, and at a time when they are less able to bear them, and even a "grasshopper is a burden."

### THE MOTHER'S REMORSE.

THE child was so sensitive, so like that little shrinking plant, that curls at the breath and shuts its heart from light. The only beauties she possessed were an exceedingly transparent skin, and the most mournful blue eyes. I had been trained by a stern, strict, conscientious mother. I was a hardy plant, rebounding at every shock; misfortune could not daunt, though discipline tamed me. I fancied, alas! that I must go through the same routine with this delicate creature; so one day when she had displeased me exceedingly by repeating an offense, I was determined to punish her severely. I was very serious all day, and on sending her to her little couch, said:

"Now, my daughter, to punish you, and show you how very, very naughty you have been, I shall not kiss you to-night."

She stood looking at me, astonishment personified, with her great mournful eyes wide open. I suppose she had forgotten her misconduct till then; and I left her with big tears dropping down her cheeks, and her lip quivering. Presently I was sent for—"O mamma! you will kiss me; I can't go to sleep if you don't," she sobbed, every tone of her voice trembling, as she held out her hand.

Now came the struggle between love and what I falsely termed duty. My heart said, give her the kiss of peace; my stern nature urged me to persist in my correction, that I might impress the fault upon her mind. That is the way *I* have been trained until I was a submissive child, and I remember how often I had thanked *my* mother since for her straightforward course. I knelt by her bed, and whispered, "Mother can't kiss you, Ellen," though the words seemed to choke me. Her hand touched mine; it was very hot; but I attributed it to her excitement. I blamed myself, as the fragile form shook with suppressed sobs; and saying, "Mother hopes Ellen will mind her better after this," left the room for the night.

It might have been about midnight when I was awakened by the nurse. Apprehensive, I ran to the child's chamber. I had a fearful dream. Ellen did not know me. She was sitting up, crimsoned from the forehead to the throat, her eyes so bright that I almost drew back aghast at the glance. From that night a raging fever drank up her life—and what do you think was the incessant plaint poured into my anguishing heart, "Oh! kiss me, mother, do kiss me. I can't go to sleep. You'll kiss your little Ellen, won't you? I can't go to sleep. I won't be naughty if you'll kiss me. Oh! kiss me, dear mamma. I can't go to sleep."

Holy little child, she did go to sleep one gray morning, and never woke again—no, never! Her hand was locked in mine, and all my veins icy with its gradual chill. Faintly the light faded out in the beautiful eyes—whiter and whiter grew the tremulous lips. She never knew me; but with her last breath she whispered: "I will be good, mother, if you will only forgive me."

Kiss her! God knows how passionate and unavailing were my kisses on her cheek after that fatal night. God knows how wild were my prayers, that she might know if only once that I would have yielded up my life could I have asked forgiveness of that sweet child.

Well, grief is unavailing now. She lies in her little tomb; there is a marble urn at her head, and a rose-bush at her feet—there grow sweet summer flowers; there waves the gentle grass; there birds sing their matins and vespers; there the blue sky shone down to-day, and there lies the freshness of my heart.

Parents, you should have heard the pathos in the voice of that sad mother as she said: "There are plants that spring into great vigor if the heavy pressure of a footstep crush them; but oh! there are others that even the pearls of the light dew bend to the earth." Mothers and fathers, be kind to the little ones. Do not wait till the daisies grow over their bosoms, before you learn to chide them in love. Kiss them before you strike them. By and by you must leave them; but leave no thorns in their memory.—

